# Psychology of Kant
*"Immanuel Kant's sämmtliche Werke," herausgegeben von Rosenkranz und Schubert. Leipzig.


**Published:** 1858-07-01

**Authors:** 


### Art. VII.—PSYCHOLOGY OF KANT.*

BY PROFESSOR HOPPUS.

The works of Kant mark the date of the second period of the German philosophy. He was born at Königsberg, in 1724; and here he lived and died, never having travelled more than a few miles from his native place. Madame de Staël remarks that there is scarcely an example, except among the Greeks, of a life so strictly philosophical. A more recent writer observes that he was a genuine "type of the German Professor: he rose, smoked, had his coffee, wrote, lectured, took his daily walk always precisely at the same hour. The cathedral clock, it was said, was not more punctual than Herr Kant."† His almost recluse life may excuse us from entering into its details, and this will better suit our space. We may, however, refer to Borowski, Schubert, and the "Biographie Universelle;" also to Jachman's "Letters." Kant died in 1804. He wrote a great number of works. The most important are his "Criticism of Pure Reason," "Criticism of Practical Reason," and "Criticism of Judgment." It is to the first of these works‡ that we shall chiefly confine ourselves.

We must not be led into any detail of the state of philosophy in Germany when Kant arose: suffice it to say that, until he had fairly grasped the sceptre which had fallen from the hand of Wolf, a sort of Eclecticism prevailed, in which heterogeneous elements were not seldom blended,—rationalism, empiricism, dogmatism, scepticism; but the predominant tendency was towards a kind of experimental psychology, and towards the history of philosophy, a source of psychological criticism which was somewhat neglected by Kant himself. The Kantian metaphysic ultimately triumphed, to give way in its turn to the other ever-varying systems which have succeeded each other in Germany; to which systems, however, Kant's writings form a clue which is wholly indispensable.

Our author was led to his speculations by the account which Hume had given of the nature of human knowledge, and more especially of the doctrine of causation. Hume maintained that our notion of the necessity of causation has no basis deeper than association and habit. Kant regarded this notion as an essential dictate of the understanding, the necessary result of our mental constitution. He tells that he soon found that the connexion of cause and effect was by no means the only one by which the

* "Immanuel Kant's sämmtliche Werke," herausgegeben von Rosenkranz und Schubert. Leipzig.

† Lewes's "Biographical History," iv. 90.

‡ "Kritik der reinen Vernunft."

understanding represents to itself, *à priori*, a necessary connexion of things; but that the whole of metaphysics consists of nothing else than such conceptions. He was led in this way to his entire system of subjective *à priori* knowledge. We may here remark, that, in pursuing his speculations, his style and phraseology are often crabbed, scholastic, and involved. It is not very often illuminated by examples, of which there is in his writings a great dearth, so that almost everything is presented in the most abstract form. The main subject not seldom seems lost in a labyrinth of entanglements. The "Criticism of Pure Reason," a work of 800 pages, sadly wants condensation. Kant tried to meet the outcry about its obscurity by writing the "Prolegomena;" in which he is certainly more brief, but not much more luminous.

Kant proposes to submit to criticism the powers and limits of the human mind. His theory is that our speculative knowledge is wholly subjective—of object or thing in itself we know nothing, but only its phenomena or appearances as presented to us. He assumes as beyond a doubt that experience is the occasion of all our knowledge. He also lays down the fact of consciousness, as at the basis of all our psychological phenomena. Here he agrees with Descartes, and his aim is the very same as that of Locke, so far as that he seeks for truth by an analysis of consciousness and a survey of the capacities of the human mind. The sense of self, the "I think" (*Ich denke*) accompanies every act of knowing; that is, we are conscious of it. Moreover, he adopts as a whole the ordinary principles of formal logic, as being an expression of the laws of thought; hence some knowledge of logic is essential to the student of Kant. We know by means of sense, understanding, reason; but we only know subjectively and in relation to our faculties. External objects are, to us at least, only phenomena appearing in space, which though itself unperceived, underlies them all. It is the condition of all outward perception, but not of the object itself. Time is the condition of all perception of things outward, and of all internal consciousness. Time, like space, belongs not to things, but only to our mode of viewing them. Space and time are the mould in which the mind casts all the objects which it contemplates. They are, in fact, mental "receptivities;" faculties by which we receive knowledge. The understanding prescribes laws to nature, and to the whole circle of our knowledge: nothing can present itself to us as a thing to be known, except in immediate subordination to the primary elements of our intellectual constitution, the conceptions of the understanding. Reason, as distinct from understanding (as Kant makes it) is deceptive as a source of speculative or scientific knowledge; for in its effort to grasp the supersensible, it breaks down, for the very reason that it aims to transcend the sphere of sense, the only proper sphere of the understanding. But when reason is *practical* (moral,) it may then attain to the knowledge of the great truths,—a moral law written on the heart, a moral lawgiver (God,) and the immortality of the soul.

We now proceed to a more special analysis of Kant's metaphysical speculations, as nearly in his own language as may consist with our being understood by the intelligent English reader; and we shall use for this purpose all the sources which his writings afford, adding, where it seems desirable, illustrations as well as explanations of our own. "He made THREE FACULTIES of the soul," the faculty of knowing (*das Erkentnissvermögen*); the æsthetical faculty, by which the sentiment of taste is gratified or pained (*das Gefühl der Lust und Unlust*); and the faculty of "appetition" or will (*das Begehrungsvermögen*). He adds, "I have expounded the principles of the first," the knowing faculty, "in the 'Criticism of Pure Reason;' those of the third," the will, "in the 'Criticism of Practical Reason.'"* He subsequently published his principles of Æsthetics in nature and art, and of Teleology, or the judgment we form in regard to the harmonies and adaptations of the world of nature, in his "Criticism of Judgment."

Agreeably to the above view of the powers of the human mind, Kant regards philosophy, in its immediate relation to the cognitive or knowing faculty, as THEORETICAL. The object is here to inquire into the laws of sense and intellect, and to decide upon the extent and validity of our speculative knowledge. PRACTICAL Philosophy seeks to determine duty, moral law, and their results; and to this branch belong the truths of man's freedom and immortality, and God's existence, all of which Kant firmly believed, in opposition to atheism, scepticism, pantheism, materialism, and fatalism; but which momentous convictions he strangely, as we shall see, pronounced utterly beyond the province of *theoretical* knowledge. Intermediate between the theoretical and the practical he places teleology and æsthetics, and regards them as belonging to that function of the cognitive faculty which we term judgment. Each part of philosophy is treated by our author as founded on *à priori* principles, that is, on those self-evident elements of knowledge which are constitutional to the mind itself, though they are all brought to notice in our consciousness only by experience: thus, from our sensuous experience of finite dimensions, we at once know that space itself (which underlies these dimensions) is infinite,—a proposition which we can never prove, because our experience can never test it, but which we feel it would be absurd to deny or doubt. Such a proposition therefore expresses an *à priori* or "transcendental" truth, so called

* "Brief an Reinhold," 1787; vide Reinhold's "Leben," 1825, s. 129.

because it transcends all actual experience. What Kant means by calling space the "form of external sense," we shall see in its proper place.

In Theoretical Philosophy we have to do with the question, "What can we know?" The "Kritik der reinen Vernunft," and the "Prolegomena," are wholly occupied with the answer. Empirical knowledge, or that which we gain from experience alone, however valuable, is far from being the highest kind of knowledge. Experience can only teach us what is, and what is likely to be. Metaphysical or *à priori* knowledge, wherever we can attain to it, teaches us what must be necessarily and universally. Metaphysics,* as applied to the soul or mind, is therefore not empirical, but rational psychology—a doctrine of the mind which we arrive at by pure understanding and pure reason, which are its only sources.† It is the peculiar characteristic of this metaphysical knowledge that it is *à priori* in the strictest sense. We sometimes use the term *à priori* in a lower sense. We say that it may be known, *à priori*, that an undermined house will fall: but the general principle that bodies fall to the earth when unsupported must have been previously known by experience. In Kant's language, *à priori* is applied to knowledge which is wholly independent of all experience and induction. Experience does not at all constitute or warrant this knowledge, it is only the *occasion* of its being elicited from our minds. If we had never seen the actual following of one event on another, in such a way as to get the notion of the latter being *caused* by the former, we should never have arrived at the principle that *every event must have its cause*. Yet, however large may be our experience of causes and effects, this proposition will always infinitely transcend that experience; for how can we be certain that, because we have never witnessed or heard of events without real or supposed causes, therefore no event ever happened or ever will happen without some cause, in all time and in all worlds? Still, we should think it absurd to doubt for a moment of this truth. Since, therefore, no knowledge of ours is antecedent to experience in time, all knowledge must begin with it. Thus the elements of all our *à priori* knowledge lie latent and dormant in our mental constitution till experience elicits what virtually lurks within; just as the occasion which discharges the electric spark reveals what before was latent and inert.

* Metaphysics, in the most general sense, means the science of ultimate and general principles, either relating to external things or mind. In Aristotelian phrase it was: ἡ πρώτη φιλοσοφία, the prime philosophy (Aristot. "Metaph." lib. i. cap. 1); and ἐπιστήμη τοῦ ὄντος ᾗ ὄντος, the science of being as such, or in the most general sense.—Ibid. lib. iv.

† Sie ist Erkenntniss *à priori*, oder aus reinem Verstande und reiner Vernunft.—"Prol." § 1.

Mathematical science furnishes brilliant examples of *à priori* knowledge, whether we consider the axiomatic truths and the definitions on which it rests, or the vast logical deductions which are made from them. Every mathematical proposition is an *à priori* judgment, based ultimately and in the last analysis on purely *à priori* principles. And here we see clearly that not only in judgments, but also in conceptions, an *à priori* origin is manifest. Thus, not only is the axiom, *two straight lines cannot inclose a space,* in the highest sense *à priori,* being both self-evident and incapable of proof by deduction from any more general *à priori* principle; but the notion of *space* itself is *à priori*—it is already pre-supposed as the necessary condition of the sensible world. We may, indeed, fancy all that occupies space, including bodies themselves, annihilated; but space itself we cannot imagine by any possibility destroyed. So we cannot but think of *substance,* corporeal or incorporeal, as something different from the properties which we experience in objects; and we cannot, by omitting this, that, or the other property, get rid of the conception of substance, which still forces itself upon us, of necessity, because it has its seat in our faculty of cognition *à priori:* we are compelled by our mental nature to pre-suppose substance as the substratum of all properties. Further, according to Kant, knowledge *à priori,* as thus explained, may be "pure" or "impure": the proposition, *infinite space is eternal,* is *à priori* and pure, as no empirical element is contained in it, for experience can never teach us either the infinity or the eternity of space: but *every change has a cause* is "impure," because the conception *change* is wholly derived from experience, though the proposition itself is *à priori.**

Immediately connected with the above distinctions between *à priori* and empirical knowledge, is Kant's distinction of all judgments into *analytical* and *synthetical*—a distinction which relates to the office and value of the predicate in any given judgment or proposition. Analytical judgments are merely explanatory (*erläuternd*); the predicate here adds nothing to the subject, but merely analyses what was already involved in it. If I say *all bodies are extended,* I have not at all enlarged my previous conception of body, I have only analysed it; for the very conception of body implies extension, and did so before the judgment itself was uttered. Now, it must be observed that in all analytical judgments the predication is *à priori,* they are mere necessary developments of their respective subjects; and this although even both the subject and the predicate may be "impure," or, in other words, wholly empirical. The term *gold,* for instance, expresses a conception entirely framed by our

* Vide "Kritik der reinen Vernunft," Einleitung, 2te Ausg.—"Prol." § 1.

experience of nature and the arts; the term *yellow,* and the term *metal* equally stand for what we have become acquainted with by experience; and the term "gold" includes them both in its meaning. Hence, if I say, *gold is a yellow metal,* I simply unfold what was already wrapped up in the term "gold:" I require (says Kant), in order to know this proposition, no further experience beyond my conception of gold, which conception contains, that it is a metal, and yellow. It is evident that the characteristic "*à priori*" cannot be generally applied to these "analytic judgments" in the sense previously laid down by Kant, but only in a much lower sense; and we think that Kant should have said as much; for surely (to take the last example) all we know of "gold" is exclusively from experience; and he says that, when we know what it is, we know the predicate of the example; yet he defines knowledge *à priori* such knowledge as is absolutely independent of all experience.* The judgments which Kant distinguishes as synthetical, are not merely explanatory, like the analytical, but augmentative (*erweiternd*); they do not merely analyse the subject by linking it with the predicate, but they add in the predicate something not contained in the subject. Of this kind of judgments are such as: *some bodies are heavy,* and (to use a former example) *two straight lines cannot inclose a space.* In both these cases, the subjects do not necessarily contain the predicates: we might attach a correct meaning to the word body without being obliged to think relative weight as between different bodies: we might have a true notion of a straight line or lines, and yet not think of them in reference to an area or bounded figure.

But while the two last examples are synthetical, in distinction from analytical judgments, they differ inasmuch as that the former is *à posteriori,* and the latter *à priori.* We will repeat these two last examples, for there is perhaps an advantage, when it is practicable, in viewing the same examples in different lights. Let us take the first, which is *à posteriori;* for how do we know anything about the weight of bodies? surely only by experience, which here enables us to add by predication something not contained in the meaning of the subject "bodies." All such propositions, then, are synthetic judgments *à posteriori.* The second example is synthetic, and it is also *à priori:* for here something is added which is not contained in the conception "two straight lines," as they can be thought without any reference to what they may or may not inclose; and we never can give any general proof from experience that "two straight lines cannot inclose a

* Wir werden also im Verfolg unter Erkenntnissen *à priori* nicht solche verstehen, die von dieser oder jener, sondern die *schlecterdings* von aller Erfahrung unabhängig statt finden.—"Vernunft-Kritik," supplem. iv. 2. Rosenkranz.

space." Further, our philosopher holds that not only all geometrical, but also all arithmetical judgments are synthetical *à priori,* for they add new matter to the subject, and carry with them necessity, which is non-empirical. Kant admits that the identical equation $7 + 5 = 12$ looks like an analytical proposition. Can I have the conception $7 + 5$ without having that of 12, and the relation of the latter term (the predicate) to the subject? He would say that I can, notwithstanding the material objective equality of the two conceptions. Our *cognising* that equality is another affair, and is subjective. We may see this if we try large numbers, when the two sides of the equation are not at once recognised as equal. Take $29,897 + 98,686 = 128,583$: here the thought of the two numbers which form the subject of this materially identical proposition, and the bare thought of their addition, do not involve at the same time the thought of their sum; we must actually add them to obtain this. It may be admitted that the theory seems at least pushed to its extreme limits in such examples as these; and if they can be accounted as subjectively synthetic, they are so, evidently, in a very different sense from that in which those examples are, on which Kant lays the greatest stress, and which lie at the very root of his entire metaphysical system — those examples, we mean, in which the predicate can in no possible way be thought out or worked out from the mere subject of the proposition—as, for instance, in the former example *every change must have its cause:* here the conception of cause lies out of the conception of change or of a mere event; it is the unknown $x$ which the understanding grasps as necessary and universal, but to which neither experience nor calculation could ever lead us.

In regard to "some few principles" which belong to mathematics, Kant somewhat modifies his theory: such are $a = a$, or the whole is equal to itself; and $a + b > a$, or the whole is greater than its part. He admits they are "really analytical." He adds, however, that they are so only by a sort of ambiguity. They, in fact, derive their validity from pure conceptions (*i.e.* by synthesis), and we regard them as having the predicate already contained in the subject (that is as analytical) only because the predicate, though truly not thought in the mere conception of the subject itself, is so thought by virtue of an intuition (*anschauung*)* which we add to the conception. Kant repeats this qualification of the theory in the "Prolegomena." It is only because these judgments can be presented in intuition that they are admitted into mathematics: and but for their being so pre-

* By an intuition, Kant understands any act of consciousness which consists of an individual object presented as existing in space or time, either to sense or imagination.

sented, they would be justly regarded as synthetical. He says that such propositions are to us analytical only because of our sensuous experience—that, for instance, our seeing wholes actually divided into parts leads us to regard the predicate "greater than a part," as contained and thought in the term "whole." We suppose Kant to mean that but for this experience the term "whole," as standing for a bare conception, would not necessarily have contained in itself the other term, "something greater than a part," in such a way as to appear only an explication of that term, and so to make the proposition analytical.

We must not omit to say that some have altogether called in question or denied the truth of Kant's theory of analytic and synthetic judgments, or, as Sir W. Hamilton would term them, "*explicative*" and "*ampliative*." M. Guiran,* for example, a strict adherent of the Hegelian school, maintains that every judgment is in itself analytical and identical, and that the distinctions of Kant are merely relative to the information of the individual. Now, if we say *a circle is a plain figure whose boundary is everywhere equi-distant from a certain point within called the centre*, this we may admit to be an analytical or identical proposition. But when we say *a circle may be drawn around any point for a centre*, it is evident that in this postulate we have gone beyond the mere definition of the circle. Again, M. Guiran's theory that all judgments vary only with the information of the individual, renders all our knowledge strictly empirical: but how can any experience tell us that *space is infinite;* yet who does not know this? We are not, however, sure that Kant has not in some of his examples extended his theory of synthetic judgments *à priori* too far.

On the general distinction between analytical and synthetical judgments, Kant grounds his entire theory of speculative or theoretical knowledge. Analytical judgments are often needful as leading to clearness of conceptions; but it is on synthetic judgments that we must rest for all the ultimate principles of rational science: not on judgments of experience, though these are always synthetical; for as they are entirely the result of experience, on which account we call them *à posteriori*, they can never be attended with absolute necessity. In the judgment, *all horned animals are cloven-footed*, for instance, we could never extract from the subject "horned animals" the predicate "cloven-footed." We learn the fact from experience, and we then make a synthesis (combination) of the predicate and subject. But though we thus generalize, we are prepared to suppose that further experience might possibly show exceptions to the rule.

* "Mémoire sur la Philosophie Allemande," 1845.

Not so in synthetic judgments *à priori;* for in these we annex the predicate to the subject with the conviction of their belonging to each other universally and necessarily: we can never imagine the possibility of any exception to the principle that *every event must have a cause.*

All the theoretical sciences contain synthetical judgments *à priori* as principles. The question, therefore, is—*how these judgments are possible?* in other words, how is pure rational knowledge possible? Analytic propositions are possible, being founded on the principle of contradiction: thus we cannot deny that *a circle has all its radii equal,* without virtually saying that a circle is not a circle. Synthetic judgments *à posteriori* are possible, as we know by the experience on which they are founded: thus we know that *water freezes at* 32° *of Fahrenheit.* In synthetic judgments, *à priori,* as we have already seen, we cannot extract the predicate by analysis out of our conception of the subject, nor can we get at the predicate by experience, and yet we are certain that the predicate belongs to the subject necessarily and universally, as in the above example on *causality.* How does this happen? We may reply, summarily, by the constitution of the human mind: but the complete solution, in detail, is the object of the Kantian critical or transcendental philosophy—called critical, because it is founded on reason's criticism of the powers and limits of our cognitive faculties—and transcendental, because it aims at bringing out the validity and the boundaries of all the knowledge we can have which transcends our actual experience.

Hence our author divides what he terms the main general transcendental question (*allgemeine transsendentale Hauptfrage*) into four parts:*—how are pure mathematics possible?—how are pure physics possible?—how is metaphysic in general possible?—how is metaphysic possible as science? Mathematics are possible because they rest on the basis of space and time, out of which all the conceptions of pure mathematics (geometrical and arithmetical) are constructed *à priori:* for space and time are *forms* of that part of the cognitive faculty which we call sense; so that we are able to have intuitions (*anschauungen*) *à priori,* by means of which we attain to the corresponding mathematical judgments. This will be better seen presently, when we come to speak of Kant's remarkable theory of space and time, which is fundamental to his system. Pure physics are possible also, because they contain certain universal and necessary principles. Nature is to us only the existence of things as determined by general laws, and these laws Kant maintains are to us wholly subjective, they are the laws under which alone we can have intui-

* Vide "Prol." § 5.

tions (perceptions) of objects, or conceptions of them. He gives as an example of a pure *à priori* synthesis in natural philosophy, *substance remains permanent—i.e.*, amidst all changes of phenomena. Metaphysic is possible, just because synthetic judgments *à priori* are possible. Metaphysic consists properly of pure *à priori* knowledge, and such knowledge we find lying at the basis of all the rational sciences. In treating of the question, whether metaphysic as a science is possible, our philosopher thus replies:—In order for metaphysic to be a science, possessing self-evident transcendental truth, its foundation must be laid by first exhibiting *conceptions à priori* (as distinguished from judgments). These must be separated, and analysed, according to their different sources in sense, understanding, and reason, and complete tables made of them, which will contain *time* and *space* as the forms of sense, the *categories* as the forms of understanding, and the *psychological, cosmological*, and *theological ideas* as the forms of reason. Such a *Kritik*, says Kant, must expound in detail all that can be inferred from these *à priori* conceptions, and must establish from their deduction the possibility of synthetic knowledge *à priori*. It must also fix the boundaries of their use, and thus we shall have the basis of a science of such *à priori* knowledge. We shall see that this science has to do, not with the objects of reason, but with reason itself merely. Yet it is not the mere anatomy of our conceptions which belongs to empirical psychology, but metaphysics proper which aims at *à priori* knowledge synthetically.

From what has preceded, Kant concludes that there results the "idea of a particular science, which may be called the Criticism of Pure Reason," since reason is the faculty which furnishes us with the above principles of knowledge *à priori*. He does not, he says, propose an Organon, or complete system in all its range, but a review of the resources and limits of reason, a kind of preliminary guide (*Propädeutik*) to a full system. Hence he prefers the name "Transcendental Criticism" to Transcendental Philosophy. His aim, he says, is rather negative than positive; he wishes to purify our reason from error, rather than to build a fabric by its aid. He seeks here to lay the foundation. He hoped to be able to carry out his deductions to a complete system, under the title of "Metaphysic of Nature." This work was not to extend to half the length of the "Kritik der reinen Vernunft," and would be to him, he says, an amusement rather than a labour. Unfortunately it was never published. In the meantime, the present "Kritik" is to contain a complete list of principles for the construction of such a work.*

* "Vorrede zur ersten Auflage," s. 14. Rosenkranz.

Preliminarily to his division of the subject of his "Kritik," our author remarks that there are two sources of human knowledge, sense (*Sinnlichkeit*) and understanding (*Verstand*). By sense objects are *given* to us; by understanding they are *thought.** Sense, as well as understanding, belongs to the Transcendental Philosophy, so far as sense can contain representations (*Vorstellungen*), *à priori,* as the conditions under which objects are given to us: for, by means of these conditions—namely, space and time, sense furnishes us with knowledge which, though manifested on occasion of experience, transcends its actual sphere, which is always limited to the particular and contingent, never embracing the universal and necessary.

We now proceed to Kant's general division of his great work, the "Criticism of Pure Reason," which is as follows:—I. Transcendental Doctrine of Elements (*Transscendentale Elementarlehre*), which occupies three-fourths of the whole volume. Under this head, we have—1. "Transcendental Æsthetic." We may observe that the word "*æsthetic,*"† here, has no meaning in common with the criticism of taste, by which we take cognizance of the sublime and beautiful. What Kant here propounds is, as we shall see, the doctrine of sensuous *perception* on his transcendental principles. 2. "Transcendental Logic;" under which head we have the two topics: (*a*) "Transcendental Analytic;" (*b*) "Transcendental Dialectic." II. The Transcendental Doctrine of Method (*Transscendentale Methodenlehre*). This is divided into four parts; namely: the *Discipline,* the *Canon,* the *Architectonic,* and the *History* of Pure Reason.

The first part of the Elementary Doctrine is termed TRANSSCENDENTAL ÆSTHETIC. The English reader must not be too much alarmed at Kant's strange and scholastic phraseology: it is, after all, generally intelligible. Our author here deals with the passive faculty of sense, by which alone objects are given to us. Sense is the faculty of *intuitions.* We have an intuition of an object when it is presented directly, in any way, to any of our senses or to our imagination—for Kant makes imagination sensuous, not intellectual. Our intuitions are of *phenomena,* of things as they seem: of things as they are (*Ding an Sich*) we can know

* Kant says nothing, at the outset, of the essential distinction which he subsequently makes between Understanding and Reason (*Vernunft*), which will appear in its proper place. We need only say, just at present, that when this distinction is not immediately in his view, his use of the term "Reason" is found in the vague, general, and diversified senses in which it has been employed in ancient and modern times. Even the term idea (*Idee*), which is afterwards exclusively appropriated to Reason (in distinction from the term conception [*Begriff*], which is appropriated to Understanding), is repeatedly employed, previously, in the lax popular sense.—Vide Krit. d. rein. Vern., Einleitung, i. ii. s. 17. 26, 27. Rosenkranz, 1838.

† *αισθανομαι,* to perceive; *αισθησις,* perception: hence the ancient distinctions *αισθητα,* things perceived; *νοητα,* things thought.

nothing, though these objective realities do exist. We only know their manifestations; that is, we know them only as they are to us. When an object produces a sensation in me, that of sound, for instance, or of vision, what corresponds in the phenomenon to my sensation is the "*matter*" of the phenomenon. This matter, then, is given *à posteriori*, or by actual experience. But phenomena must present themselves to sense, also, under a certain "form." Let us separate from our representation of body all the conceptions which the understanding annexes to sense—such as those of substance, force, etc.; and having thus isolated the sensuous faculty, let us suppose taken away from our intuition all that is empirical—all that belongs to mere sensation—as colour, hardness, solidity, &c., and what remains? only the *form*, or pure intuition, which is all that sense can give us, *à priori*. Now, says Kant, there are two pure forms of sensuous intuition—namely, Space and Time. *External sense* places before us objects as without us, and always in space, in which alone shapes, dimensions, and mutual relations are determined. *Internal sense*, by means of which the mind contemplates its internal state—gives us, indeed, no intuition of the soul as object, as external sense gives us intuitions of body; still there is a determinate form under which alone the contemplation of our internal state is possible: all our mental processes must go on in time. We have no external intuition of time—we cannot see it (that is), hear it, or the like: nor have we any internal intuition of space, our intuition of it is wholly external. Time and space are not real things—they are not relations of things: what, then, are they? They belong only to us subjectively: they are, in fact, properties of our minds.

Space is not an empirical conception (Begriff), that is, one which is derived from external experiences: for I am always obliged to presuppose its existence as the very background of all my experience of objects. It is the condition which renders phenomena possible. Imagine a new orb created—space must already be in its place. Imagine all nature annihilated—space still remains. Space, then, is a representation *à priori*—a pure intuition; it is prior, that is, to all phenomena, and is pure from, or independent of, all our empirical knowledge of given dimensions: for they only overlie portions of its all-comprehending infinity. No mere conception can reach this infinity: yet every part of space may be infinitely produced. Hence, though it gives rise to conceptions, its original representation is intuition *à priori*—not conception. Further: Kant argues that our representation of space must be originally intuition—that is, an affair of sense, because we can never get out of any mere *conception*, by its analysis, more than was before contained in it (hence analytical judgments, as above); and yet, in geometry, the science which determines the properties of space, we get propositions which go beyond the subject of them, and obtain predicates, such as to make them synthetic and *à priori*. For this intuition of space stretches far beyond all experience, and underlies all perception of objects, showing that it is independent of them, and so rendering geometry possible, and its principles necessary. Kant gives us examples of such a principle: "*space has only three dimensions*:" "*different spaces are not successive, but co-existent*." And how, he asks, "can an external intuition anterior to all objects, and in which our conception of things can be determined *à priori*, exist in the human mind?" Only so far as it is native to the mind itself, having its seat only in us the subject; only inasmuch as it is our capacity of being affected by objects.

From these conceptions which we are obliged to form of space, Kant concludes that space is only the "form of external sense," or the *subjective* condition under which alone perception is possible. In regard to our cognizance of phenomena, or things as they appear, space has objective validity, for everything presents itself to our senses as in space. It has, therefore, so far empirical reality; but space is nothing the moment we regard it as belonging to things in themselves—that is, it is merely ideal; for things in themselves are to us a blank, we know nothing of them. What we call outward objects are truly nothing but mere representations given us by our sensuous faculty, of which space is the form; but the correlates of the phenomena thus represented, or the things in themselves, we can never know by sense; and as we have no *intellectual* intuition, (whatever any other beings may have,) we cannot know them at all. In one word, according to Kant, space is *in us*—not in the universe around us: it is a condition of the exercise of our sensuous faculty—not a condition of real objects. Yet, strange to say, Kant strenuously maintained the real existence of material objects as the basis or substratum of the phenomena which these objects occasion in us, while he would not allow it to be said that the real object requires space to exist in! It is no wonder that in discussing this subject, Kant's language is perpetually at war with his subjective theory—just as Berkeley cannot help talking of matter as a real thing every moment, while he denies its existence, and pronounces it an impossibility. It seems obvious enough, surely, that if material objects really exist independently of us, as Kant said they did, space must be as much a necessary condition of their existence, as it is a necessary condition of our perception of the phenomena which belong to them.

We must not omit a curious illustration which occurs in the

"Prolegomena,"* brought forward as a convincing proof of the ideality of space as being a mere form of perception; in other words, a mere function of our sensuous faculty, having no true externality, but being exclusively in us. The glove of one hand cannot be used on the other: the two objects are similar and equal, but not congruent—why so? "because space is nothing but a determination of our sensitive faculty." A more singular argument, surely, can hardly be imagined. We should rather say that the case was an illustration of the real externality of space: for it is evident that when the left-hand glove is reversed, the separate four fingers each fit those of the right hand well enough, but the thumb is awkward because it is now pitched backwards instead of forwards. It is extraordinary what a passion for a theory will do! We confess that if such an argument has any meaning, to us it seems to go against the ideality of space rather than in its favour. The same may be said of two other illustrations which Kant brings forward—the incongruency of the equal images of the hand in a mirror—and the incongruency of equal spherical triangles on the globe, one in each hemisphere, when the triangles have for their common base an arc of the equator.

TIME, adds our author, is also not a conception which is drawn from any experience: for neither co-existence nor succession would come into perception,† if the representation of time did not lie *à priori* as a foundation. Things could never seem to us contemporaneous or successive, unless time for them to exist in, either together or one after another, were presupposed. We cannot think away time from phenomena, we must always perceive them in time: it is the universal condition of their possibility, though we may imagine time void of all phenomena. The empirical conceptions of change, and therefore of motion, are only possible through and in time; and from this necessity of time as the indispensable condition of these conceptions, and of all phenomena, Kant terms it an internal intuition *à priori*, and the *form of internal sense*, as space is the form of external sense.

On this necessity is founded the possibility of synthetic judgments *à priori* (as before explained) in relation to time: such as *time has only one dimension* (linear, or that of continuous progress); *different times are not co-existent but successive*. These axioms cannot be derived from experience, which can never give either absolute universality or absolute necessity to our knowledge. Such axioms in fact give law to experience, and render it possible. They are the results of the pure form of our sensuous intuition *à priori*. Such is another example: *different times are*

* Sect. 13.

† *Wahrnehmung*—Kant sometimes uses this word as synonymous with *Anschauung* (intuition).—Vide "Prol." § 10: "Anschauung, d. i. Wahrnehmung wirklicher Gegenstände."

*but parts of one and the same time.* Again we could not say *time is infinite,* unless we had the original unlimited representation of time in us as a basis. We may here remark, in passing, that, to us time seems more perplexing than even space. Kant has not remarked this. Indeed, his wholly subjective views probably prevented him from seeing the difficulty we allude to. Even Kant, however, notwithstanding his idealism of space and time, cannot avoid speaking of them objectively. Now we feel able readily to imagine space as wholly denuded of all objects—in fact, as an infinite void; but can we so readily represent to ourselves, as our author says, "time void of all phenomena?" The pure intuition (as Kant calls it) of time, is not more *à priori* on his system, than the axiom "time has only one dimension." We see well enough what he means by this, for time has only length: but can we represent time to ourselves at all, excepting as a kind of flow? A flow of what?—of changes surely—of successive phenomena. How do we know that time has "only one dimension," but by our being quite unable to represent it to ourselves otherwise than as marked by perpetual progressions or successions? Grant that successions are in time—yet, again, what is time apart from all successions? It will be seen that we here hazard no theory of time: we only start a difficulty—perhaps an objection to Kant's statement "that we can very well represent to ourselves empty time."

Our author further remarks that space, being the pure form of *external* intuition, can only be the condition of external phenomena. That is, space is not the condition of thought. Time, however, is the pure form *à priori* of all phenomena, whether of nature around us, or of mind itself. When he calls time the form of the *internal* sense, he means that it is the *à priori* condition of "the intuition of ourselves and of our internal state."* Time is the *immediate* condition of all internal, and thereby the *mediate* condition of all external phenomena. It will be observed that Kant makes time as well as space sensuous: for man's intuition, he says, is always sensuous. I can only "*intuit*" (have a sense of) myself through this form of internal intuition. It is true enough, as Kant says, that there is a difficulty, common to every theory, in saying—how the subject or *ego* (the *me*) can have an internal intuition of itself. The reflex act of self-consciousness—the cognizance we take of self—the introversion, as it were, of the mental eye upon itself, in our being conscious of our own personal psychological phenomena—this is a *fact,* however inexplicable. But it is quite another thing to say that our consciousness of self is a sensuous, not an intellectual pheno-

* Des Anschauens unserer selbst, und unsers innern Zustandes.—"Kritik d. reinen Vernunft," s. 42. Rosenkranz, 1838.

menon: this, however, is what Kant says, and it is a peculiarity of his doctrine of Transcendental Æsthetics. His argument is, that the representations of consciousness, in the *ego*, the conscious subject, are given without spontaneity—that is, passively: hence, consciousness is wholly an affair of the sensuous faculty. On this principle, our consciousness of thoughts is as sensuous as our consciousness of sensations, or of the intuitions and perceptions of which sensations are the "matter."

Our philosopher, before closing his remarkable theory of the absolute ideality of time and space, and of the wholly subjective character of all that belongs in any way to sense, specially guards the reader against supposing that his theory of the subjectivity of all phenomena, as essentially connected with that of the subjectivity of their pure or *à priori* forms (time and space,) by any means involves the assertion that phenomena are mere illusive appearances. No; objects as phenomena are really given; but as they are only given relatively—that is, so far as they are related to the conscious subject—we must distinguish the object as phenomenon, from the object as a *thing in itself.* Kant maintains very strenuously, in this part of his subject, that everything would truly be changed into mere illusory appearance, if we regarded space and time as objective—that is, as having any functions or relations out of our minds; though he does not make very evident how he deduces such a conclusion. He holds, too, that even our own conscious existence would become a mere illusion if we made it to depend on the objective reality of time, which, like space, can only be a form of the human mind, which, as a mould, gives its own shape and figure to what is applied to it.

The above is Kant's doctrine of perception: let us sum it up. What we call objects, are only revelations of them as they appear to our sensuous faculty. The real object we know not. What is variable in our sensations, varies with the agency which the objects exert on us; but there are two invariable elements which attend all our sensuous experience—space and time. Everything without us presents itself as in space: everything without us, and all our inward consciousness, are presented to us as in time. The reason is, that space and time are furnished by the mind itself. They have no existence apart from the mind: they do not adhere to the real objects themselves, as is commonly supposed. Time and space are not in the universe; they are only in us: they are essential constituents of our sensibility, or sensuous faculty; they are the forms or modes under which external objects and our own mental processes present themselves to us. As our knowledge of external objects is thus only phenomenal, not real or substantial,—so our knowledge of ourselves, our souls or minds: our inward consciousness only presents to us the *me*, as phenomenon—that is, as it appears, not as it really is. It will be seen at once how Kant's idealism of perception differs both from Berkeley's and Fichte's: from Berkeley's, in holding that there are in the universe positive, real things apart from our minds, though we know nothing more of them, in themselves, than though they existed not; while Berkeley maintained that there was nothing but minds or spirits—all that seems real in nature being only modifications of our minds: from Fichte's, in holding that the mind is passive in sensation, being acted on by an outward *non-ego*, or not-self; while Fichte said that the mind unconsciously and spontaneously spun from itself the whole universe, and then mistook it for a reality. It is worth remark, that Kant pronounced Berkeley's idealism "fanatical,"* (calling his own "critical," and "transcendental;") and Fichte was named in Germany "the consistent Kant," because he boldly accepted the consequences of Kant's denial of all objectivity to time and space, and went the whole length of idealism.

No doubt, time and space are, as our author says, conditions, the one, of *all* our psychological phenomena, the other, of all the psychological phenomena of *sense*. Time, to us, underlies all thought and all perception: we can think and perceive only in time. Space, to us, underlies all perception: we cannot perceive any external phenomenon, or even imagine it, but as presented in space. But is this all? "Yes," says Kant, "space and time have no connexion whatever with things themselves—the realities which present to us the phenomena. Space and time are wholly in us." This doctrince is, we hold, perfectly gratuitous, and we may say inconceivable, if there be, as Kant says there are, real things. We may admit with our philosopher that all our knowledge is relative to our faculties. Things are known to us only as they appear to us; we only know substance as that which we cannot but suppose is the basis of the properties which appeal to our senses. We cannot know substance in itself, for we cannot imagine it apart from its properties, nor can we know the properties but as properties of substance; we cannot conceive of external or internal phenomena as the ghosts of nothing—we can only apprehend them as phenomena of matter or mind. No doubt, sensation is the result of the constitution both of the object and of the subject; just as a table is the result of the matter (wood) and the form which the workman gives to it, or as a piece of pottery is what it is both from its material and from its mould. So our perceptions of things can only be as things are exhibited to us, and as our perceptive powers enable us to receive

* . . . . mit dem mystichen und schwärmerischen des Berkeley. . . . Proleg., § 13. Anmerk iii.

them. But all this, surely, does not interfere with the objectivity of time and space. Surely it is not only of our nature to know things as in space and time, but it is also of the nature of *things,* if they exist at all, to exist in time and space. If we may say of physical phenomena that they present themselves in time and place, why should not their acknowledged causes (and Kant admits that real objects are the causes of phenomena) also exist in time and in place? And as the existence of the soul is also admitted, and its phenomena or processes go on in time, why should not the soul itself exist in time? How can the real being, the cause of the manifestations, be out of time, any more than the manifestations themselves? Surely the soul is as enduring as its phenomena; and how can either endure apart from time? How can effects be limited to time, or space, or both, and not their causes? It is quite inconceivable that a material object can produce effects where it is not present. Our not understanding the nature of substances or existences in themselves, does not at all militate against our bringing them under the time-and-space conditions, but the reverse; for all we know of them is, that they are something inseparably connected with their phenomena, and we are unable even to think of them apart from the latter. If things in themselves, or *noumena,* act at all, they must surely act somewhere—at some time. We need not say that we have empirical knowledge of the existence of real objects in time and space, as we have of objects as phenomena; but reason cannot but necessarily infer that, if they exist at all, they must exist in time and space. In this inference, reason only does just what she does in pronouncing that there are substances at all; for the conception of substance itself is not empirical, but *à priori,* being necessary by a law of our mind to the conception of properties and manifestations.

It is evident that, to have been consistent in denying the externality of time and space, Kant ought to have denied a material world; his entire subjectivity of nature would square very well with Berkeleianism or Fichteism, but with his own avowed realism it is quite at variance. He believed in the real planets; but what, on his theory, would become of their motions round the sun in space and time, supposing all sensuous faculties to cease to exist? If time and space are mere "receptivities" or forms of our minds—what if there were no sentient beings? Is it conceivable that their annihilation would annihilate the time and space in which motion alone is possible? What imaginable effect could the destruction of all sentient creatures have on the movements of the planets? Kant's admission of realism may safely be pronounced to be opposed to the whole spirit of his speculations.

Again, it is a capital error of Kant's system that it makes time and space wholly *sensuous*. No doubt, time and space are the *à priori* conditions of our knowledge—the former, of our knowledge of our mental phenomena; and both, of our knowledge of the phenomena of sense. Yet it should not be said, on this account, that our knowledge of the necessity and universality of time and space, as such conditions, is of the province of sense. True, it is our sensuous nature that enables us to receive sensations, and to receive them as the signs of the external phenomena which cause them; but though sense is the occasion of our *à priori* conceptions of time and space, these conceptions themselves surely belong to our intellectual faculty. To call them "pure intuitions," in distinction from the empirical intuitions (representations which actual experience gives us) of objects, is merely to beg the question. It might as well be said, that because our conception of *cause* is occasioned originally by our experiencing actual changes in the external world through the medium of our senses, therefore this conception belongs to sense. This, however, even Kant himself expressly denies, and affirms that our notion of causality belongs to the understanding. In fact, there is no proper distinction between this latter faculty and Kant's "pure sensibility."

We actually see limited spaces—we rise, in thought, from this sensuous experience to the conception of an infinite, eternal space, which renders all our experience of limited extensions possible; but surely this latter phenomenon requires another faculty —intellect. With regard to time, we are, if possible, still more obviously shut up to the understanding. For how can our most limited notions of time be regarded as sensuous? Here time, we cannot but think, differs greatly from space; and Kant is not, in our judgment, the only philosopher who has erred by attemping to run a forced parallelism, throughout, between time and space. Grant that our muscular sense, or our visual perceptions, primary or acquired—that, in short, our sensuous nature alone enables us in the first instance to have any *limited extension* presented to us in the concrete, as, for instance, the length of a book in our hands, or lying before us: but, on the other hand, can we say that our notion of an hour or of a minute is a sensuous phenomenon? May we not have this notion quite abstractedly from sense, by means of the mere succession of our own thoughts? May we not be looking constantly at a clock that is before us, and see the continued progress of its hands, and yet, under different circumstances, have very different notions of the lapse of time; the notion of a long time, with weary waiting; the notion of a short time, with absorbing and agreeable ideas and emotions—and yet the actual time elapsed shall be the same? True, we *measure* time accurately by numbering the visible beats of the pendulum; but can anything really be more intellectual than all our notions of time, even of its briefest intervals? Yet Kant makes time as well as space wholly sensuous, though he speaks of them frequently as "conceptions" (*Begriffe*).

More than this: Kant even attaches our *consciousness* to the faculty of sense. We not only take cognizance of external phenomena as occurring and existing in time; we are also compelled to regard all the facts of consciousness as taking place in time—this no one doubts. But Kant is hence led to make consciousness itself an affair of sense; for time is as much the form of our internal as of our external intuitions—that is, it is the form of consciousness. He first makes time sensuous, and then consciousness is sensuous too. In consciousness we have presented to us what is going on within our minds: we know our internal state by observing that it undergoes certain changes or modifications; only in this way is consciousness possible. In being affected with these modifications we are wholly passive, just as we are when affected by outward phenomena. Hence, while sight, hearing, smell, etc., constitute external sense, having space and time for its forms,—consciousness is nothing more nor less than internal sense; and Kant tries to justify his attaching it to our sensuous rather than to our intellectual nature, by dwelling upon its passive character, and denying to it all spontaneity. His argument would equally prove that our capacity of appreciating the sublime and the beautiful is sensuous, for certain affections or emotions are awakened in us by certain objects, involuntarily; yet it is only rational beings that can discern æsthetical relations, and Kant himself refers them to our intellectual faculty. If by consciousness of what passes within us be meant our cognizance of our various internal modifications as our own, surely consciousness is essentially intellectual, however accompanied or even empirically originated by sense. Even our judgments are not always voluntary: Kant himself says, that the categories according to which we pronounce our judgments are not subject to our will, but are necessary laws of thought. In thus making consciousness sensuous, we hold that our author is again placing at the basis of his system another capital error. We shall see what he says further of consciousness under the next head of the "Kritik;" and let us not be surprised if we shall find it hard to reconcile his assertion that consciousness is a modification of the sensuous faculty, with his subsequent theory in which he views consciousness in relation to the *understanding*.

We now proceed to the Second Part of Kant's "Elements;" namely, his TRANSCENDENTAL LOGIC.

"Our knowledge," he says, "is wholly by intuition, and by conception. Intuition and conception are both necessary in every case in which we can be said to *know* with the understanding. Both may be either pure or empirical. When we have an object presented to us by our receptive faculty of sense (as when we actually see or hear anything), we have an *empirical* intuition of it. When we think an object in relation in some way to its representation, we have a *conception* of it. A savage sees a distant house merely as an object, not knowing its use: he has only an intuition of it. Another person recognises it as a dwelling for man; he has in addition to the intuition also a conception, which the other has not." Kant would say, that as to the *matter* of their sensuous impressions, both were alike: both saw the object; but the *form* of the cognition is in the one only intuition, in the other both intuition and conception.* All, however, is here empirical (only through actual experience). So also the conception of *change* is empirical: to make it intelligible, we must refer it to some actual phenomenon. But both intuition and conception may also each be *pure*. We necessarily must have all external phenomena presented to us in space—space is a pure intuition, as we have already seen. Our conception of cause is a pure conception; for we cannot see causes, or have them presented to our senses: we only see changes—we *think* their causes. Sense is a passive faculty (a mere receptivity): understanding, by which we can have conceptions, is a spontaneous faculty; without sense, no object would be given: without understanding, no object would be thought. Thought without some object to think of, is void: intuition, or any sensuous representation, without conception, is blind. It will here be borne in mind, that it is a peculiarity of Kant's theory, that we can have no conceptions, whatever, but such as have a relation to sensuous objects. Of this more hereafter.

Pure logic is strictly formal, relating only to the form of thought, and apart from the matter or subject of thought. This alone is properly the Science of Logic. By *Transcendental Logic,* our author understands an inquiry into the origin and validity of the pure forms of the understanding. There are intuitions *à priori,* the necessary forms under which all sensible objects must be presented to sense: these are time and space. There are also conceptions *à priori,* the necessary forms under which all thought which relates in any way to these objects, must occur in the understanding. Now, Transcendental Logic is the pure science of these laws in their origin: it is an exposition of the source and basis of Universal Logic.

* Vide Kant's "Logik," Einleitung v.

Logic, whether general or transcendental, may be divided into *Analytic* and *Dialectic*. Analytic unfolds the elements of the function of the understanding, and comprises the necessary laws of all formal truth—truth being the accordance of our knowledge with its objects. Still, Analytic only furnishes a negative test of truth, a *sine quâ non:* it furnishes no test of the matter of our knowledge. Grant that if there be a painting which could only have been painted by one out of four given painters; then Logic will tell you that if it was not painted by A or B, it must have been painted by C or D; but Logic will not tell you by which. By *Dialectic,* Kant (agreeably, as we shall see in the sequel, to his peculiar theory of understanding and reason) means the erroneous application of the analytical or formal Logic to our knowledge, by way of deciding on its objective truth—its application to things which Logic can never teach. From this practice, all sorts of delusions arose among the ancients and the scholastics. Hence, according to Kant, Dialectic is a "logic of mere appearance" (*ars sophistica disputatoria*). In the *Transcendental* Logic, Analytic inquires into the elements of *à priori* knowledge in the understanding—the forms with which the understanding must clothe all the intuitions of sense, and the principles without which no object can be in any way thought. He here expressly maintains that we can never, without illusion, apply these forms beyond experience. Dialectic, transcendentally considered, is a criticism of the dialectical illusion which arises from attempting to use the analytical elements and principles beyond the objects and limits of experience.

I. *Transcendental Analytic;* under which head are two divisions—the Analytic of Conceptions, and the Analytic of Principles. This Analytic proposes the analysis of all the *à priori* knowledge which the understanding can give us. The discussion concerns only those elements which are pure, not empirical; and which at the same time belong to understanding and thought, not to sense and intuition. All must be strictly elementary, not deduced; and the enumerations must be complete. One part of the inquiry contains all the conceptions; the other, all the principles of pure (or *à priori*) understanding.

1. The Analytic of Conceptions (*Analytik der Begriffe*). The analysis of conceptions is not resolving them (as we do complex ideas) into their constituents, and so making them clear. This Analytic relates directly to the faculty of understanding itself, and its *à priori* use. We must trace the germs of all the pure conceptions as they lie in the understanding itself, until they are elicited on occasion of experience. We must remember that the understanding is not a faculty of intuition; according to Kant, it presents no objects—only sense can do this: but its conceptions are such that sense must present objects always according to them. All that the understanding can do is to judge by means of its conceptions. A judgment is a predication, real or virtual—a saying that A is B, or is not B. All acts of the understanding are reducible to judgments, so that it may be defined the "faculty of judging." Conceptions, as predicates of possible judgments, relate to some representation of an object as yet undetermined. The conception of "body" in general, for instance, is undetermined; but it may be the predicate of a great variety of judgments; for this conception contains a great many representations under it, the very thing which makes it a conception. In this way it can relate to objects, as all conceptions must: thus we can say, for instance, *metal is body,* or *every metal is a body.*

Now, says our philosopher, if we analyse what takes place in the actual exercise of the Logical Function of the Understanding in Judgments (Propositions), we shall immediately obtain all the pure or *à priori* forms or conceptions of the understanding, just as the inquiry into our sensuous faculty gave us the pure or *à priori* forms or intuitions of sense—namely, space and time. We have, therefore, first a Table of all the kinds of judgments. Here all that relates to the matter or topic of the propositions is rejected (as all that related to the matter of the object of sensuous intuition was rejected), and only the form is retained. It will be seen that this Table takes for granted most of the distinctions of propositions as found in the common formal Logic, and adds to them some others of a psychological nature, merely, rather than logical. Now, the functions of thought, in a judgment, says Kant, can be brought under four heads, each containing three *momenta* or divisions, as follows:—

TABLE OF JUDGMENTS.

| 1. Quantity. | 2. Quality. | 3. Relation. | 4. Modality. |
|---|---|---|---|
| Universal, | Affirmative, | Categorical, | Problematical, |
| Particular, | Negative, | Hypothetical, | Assertorical, |
| Singular. | Infinite. | Disjunctive. | Apodictical. |

This Table is given as including all possible judgments. We will explain it to the non-logical student, a little more particularly than Kant has done. The *quantity* of a judgment consists in the greater or less extension of the subject of the proposition—namely, that of which something (the predicate) is said: thus, *all men are fallible,* is universal; *some men are poets,* is particular; *this man* (Mr. A.) *is a sculptor,* is singular.

The *quality* of a judgment consists in the position of the subject with regard to the predicate—that is, whether it lies within or without the sphere of the predicate: thus, each of the three above examples are affirmative; and *no men are perfect, some men are not wise, this man is not an artist,* are all negative. What Kant singularly terms the infinite (*unendlich*) judgment, is not found in the common logic. There would be less of ambiguity in calling it "*limitative;*" and this would exactly agree, as we shall see, with the category of "*limitation,*" which is at the foundation of it. This limitative judgment is one which is affirmative in form, though with a negative predicate. Psychologically there is negation, logically there is affirmation: thus, to use Kant's own example, while "*the soul is not mortal*" is a negative judgment, "*the soul is non-mortal,*" though affirmative in form, is really *limitative,* for it restricts the soul to the sphere of beings that are not mortal.

The *relation* of a judgment consists in the way in which the terms of it are connected with or subordinated to each other. In the categorical judgment, the terms are connected merely as subject and predicate, without any condition; as, *A is or is not B.* In the hypothetical (conjunctive) judgment, the terms are connected as antecedent and consequent; as, *if A is B, C is D.* In the disjunctive judgment, the relation is that of the logical opposition of two or more propositions, so far as the spheres of the propositions respectively exclude each other: but there is also a "relation of community," so far as the propositions make up, in common and altogether, the whole sphere of a cognition; as, *either A is B, or C is D, or E is F, etc.* Kant's example is, *the world exists either through blind chance,* or *through internal necessity,* or *through a cause external to itself.* Here each proposition contains a part of the sphere of our possible knowledge in regard to the existence of the world; all of them together contain the whole sphere.* To reject any one of these assumptions is to adopt one of the rest; and to adopt any one is to reject the others.

The *modality* of a judgment consists merely in the "value of the copula." In problematical judgments, a predication is made as merely possible; as, *the soul may be immortal:* in assertorical, the predication is made as actual; as, *the soul is immortal:* in apodictical, it is made as necessary; as, *the soul must be immortal.*† In an hypothetical (conjunctive) syllogism,—such as if A is B, C is D; but A is B; therefore C is D,—we have a combination of all the three kinds of modality. The antecedent, A is B, is given problematically in the first proposition (major); it is given assertorically in the second (minor); and in the third proposition, which is the conclusion, C is D follows apodictically,—that is,

* "Kritik der rein. Ver., Elementarlehre," s. 74. Rosenkranz, 1838.

† "Logik;" *von den Urtheilen,* § 30.

necessarily; for when once A is B is admitted, it follows that C must be D.*

For the sake of the non-logical and non-metaphysical reader, we volunteer two or three examples by way of further illustrating Kant's whole doctrine of judgments: *all men are mortal* is universal in quantity, affirmative in quality, categorical in relation, and assertorical in modality. *If A is B, C is D*, is (according to what A stands for) individual, particular, or universal in quantity, affirmative in quality, hypothetical (conjunctive) in relation, and problematical in modality. *No circle can have more than one centre* is universal, negative, categorical, and apodictical.

Such is our philosopher's account of the "logical function of the understanding in judgments;" which, as it is connected with the categories, and indeed with his whole theory of understanding, we have dwelt on at some length. These categories (so called from those of Aristotle) are, in fact, pure conceptions of the understanding. They are involved in the above judgments, and they render possible all our cognitions (knowledge) by synthesis,—that is, by the mental process of joining different representations in one notion. Thus, if I say, there is a *tree*, I have a notion which is formed by the putting together into one of diverse representations or qualities which go to make up the notion. The synthesis here is empirical, for I only know the properties of a "tree" by actual experience. The synthesis is pure when the elements which it unites are given *à priori;* thus our notion of a *decade* is formed by a pure synthesis of unities, and unity is one of the pure or *à priori* conceptions of the understanding. Now, says Kant, all that we can possibly say of any object that we can know anything about, we must say in one or other of the ways contained in the Table of Judgments; and from this Table we immediately obtain all the possible categories of thought, or ways in which objects can be viewed by the understanding, which by its pure conceptions must give law to all our possible experience. Hence the categories will exactly correspond with the judgments. There can be no more and no fewer ways in which the understanding can take cognizance of its objects. Kant adopts the term "categories" from Aristotle, though with an entirely subjective

* This is a case of the *modus ponens* of the schoolmen (in which the antecedent is admitted). The case of the *modus tollens* (in which the consequent is denied) would, of course, do as well for the illustration.

The logical reader will notice that, according to a strictly formal common logic, the distinction of modality, or the *matter* of propositions (which Kant has introduced into his Transcendental doctrine,) is extralogical, notwithstanding that Aristotle adopted it in his logical treatises, along with a vast mass of other extralogical things. In this he was followed by his successors. The modality of propositions was one of the most perplexing and useless disquisitions in which the schoolmen engaged. They had a saying, *De modali non gustabit asinus.*

meaning, his design being merely to exhibit the forms of the understanding, or the ways in which objects must, if given at all, be given to it. We request our readers carefully to compare the following Table with that of Judgments, when the correlation of the two will be at once evident. The conceptions which it contains are indifferently termed "categories," or pure conceptions of the understanding (*reine Verstandesbegriffe*).

TABLE OF THE CATEGORIES.

| 1. Of Quantity. | 2. Of Quality. |
|---|---|
| Unity, | Reality, |
| Plurality, | Negation, |
| Totality. | Limitation. |

3. Of Relation.

Of Subsistence and Inherence (substance and accident).
Of Causality and Dependence (cause and effect).
Of Community (reciprocity between agent and patient).

4. Of Modality.

Possibility—Impossibility.
Existence—Non-existence.
Necessity—Contingence.

Our author gives the above as a complete list of all the pure conceptions of the understanding. Only by means of these conceptions can it think any object of sense. All the conceptions arise, as we have seen, from the faculty of judgment, which Kant identifies with the power of thought.* He compares his categories with those of Aristotle, whom he speaks of as having sought for these fundamental conceptions without any guiding principle. Kant, however, here evidently overlooks the fact that Aristotle's categories were objective, his own subjective. Aristotle's enumeration is of objects and their qualities and relations as viewed by the understanding; Kant's is an enumeration of the subjective determinations of the understanding itself, in reference to possible objects.

The classes of conceptions under *quantity* and *quality* are termed mathematical; those under *relation* and *modality*, dynamical, as having correlates. The former refer to objects of intuition: empirical, as, for instance, *tree;* or pure, as *space, time.* The latter refer to the existence of objects, either in reference to each other or to the understanding. Further: in each triad, the third category arises from a combination of the other two. Thus, Totality is nothing more than plurality combined with unity; for one whole is constituted of all its parts. Limitation is only reality, joined with negation of the same. Thus, a finite

* Diese Eintheilung ist aus dem Vermögen zu urtheilen, welches eben so viel ist als das Vermögen zu denken. "Kritik," s. 79. Rosenkranz, 1838.

right line has a real length, beyond which there is a negation of further length—that is, the line is limited. Again, in the general category of Relation, we have the relation which exists between things or substances, and their attributes or accidents which inhere in them; the relation between causes and their effects which depend on them; the relation between things which reciprocally act and re-act on each other. Now, here, again, the third sub-category arises from the combination of the other two: for Community or Reciprocity combines causality (as implying the dependence of the effect) with the inherence of causality (as an attribute) in the substance; in other words, Community is the causality of anything in reciprocal determination or agency with something else. Under the fourth head, of Modality, how do we, by combining possibility and existence, obtain Necessity?* and how do we (in the opposites) obtain Contingency from the combination of impossibility and non-existence? In order fully to explain these points, we must more articulately compare the Table of Categories with the previous Table of Judgments.

The correspondence between the judgments and the categories, in the cases of Quantity and Quality, are sufficiently obvious. In Quantity, it is evident enough that *universal* propositions would not be possible without the previous (*à priori*) conception of totality—all. So, *particular* propositions, in like manner, imply the conception of a part, or a number less than the whole—some. And *singular* propositions are founded on the pure conception of unity—one, as this or that individual. We have already remarked that totality is the unity of parts or particulars. In Quality, *affirmative* propositions imply the reality or actuality of the connexion between subject and predicate. *Negative* propositions rest on the conception of negation. *Limitative* pro-

* Kant himself says: "Necessity is nothing but existence, which is given through possibility itself" (*Ed. Rosenkranz*, supp. xii.). If this means that before anything actually exists it must be *possible*, and that being thus first possible, and then actually existing, its existence has now become a necessary fact, which can never cease to be a fact, even though the existing object were afterwards annihilated, we understand the meaning. But we are told by an able student of Kant, that "his meaning is, that a necessary existence is an existence whose existence is given in the very possibility of its existence." Some of our readers will be aware of the Cartesian method of argument, borrowed from Anselm, and remodelled by Leibnitz, as follows: "God alone has this peculiar distinction (*hoc privilegio gaudet*), that if he be possible, he necessarily exists; and since nothing stands in the way of his possibility, this alone suffices for our knowing the existence of God *à priori*" (*Leibnitzii Opera, Dutens* ii. 16, 17). Now, we are not here called on to examine the validity of this argument for the Divine existence, but only to remark that it is distinctly repudiated by Kant, who says: "It is a contradiction to introduce—under whatever term disguised—into the conception of a thing which you are to think of solely as to its possibility, the conception of its existence" (*Ed. Rosenkranz*, 465). If, therefore, Kant means to illustrate the category of Modality by this example, he is using for this purpose a theory which he himself rejected.

positions have at their basis the conception of limitation. We have before seen that reality and negation combined amount to limitation.

In the Category of Relation, we obviously see the accordance of the two first sub-categories with the corresponding logical function of judgment. The *categorical* proposition pronounces that some attribute (*accidens*) named in the predicate inheres in (belongs to) the subject—as snow is white, man is an animal; for though, in the common logic, such propositions (except singular ones) are regarded as expressing classes of things, still the predicate (" animal," for instance) admits of being viewed as expressing a property—animality. In the *hypothetical* (conjunctive) proposition, we find the principles of causality and dependence, or cause and effect; for the existence of the consequent depends on that of the antecedent—as, if A is B, C is D. The *disjunctive* proposition makes its subordinate parts mutually dependent on each other, throughout: either A is B, or C is D, or E is F, etc., means that the whole complex proposition here given is divided into parts which mutually exclude each other, one position only being admitted, whichever it may be. One part is not contained in another, but they are all thought co-ordinately and separately, and determine each other mutually. Hence the basis of the disjunctive judgment is the conception of the reciprocity of certain agencies or co-ordinate positions. We have before shown how Kant makes the sub-category of reciprocity or community arise out of those containing the correlates inherence and subsistence, and causality and dependence.

Under the general head of Modality, we must premise that our author's *problematical* judgment is not peculiar: it is identical with the hypothetical (conjunctive) or the disjunctive judgment, as the case may be. His *assertorical* judgment is, in fact, categorical. His *apodictical* judgment is really the same, but he defines it as expressing logical necessity. Indeed these three judgments of modality are, as we have before remarked, strictly extralogical. The conception of possibility, of existence, and of necessity, are evidently essential, respectively, to each of these three modal judgments. It is not difficult (though Kant has nowhere logically exemplified it) to see how, if we keep close to the Table of modal judgments, we may get necessity out of possibility and existence combined. Thus, the following argument is correct; though the " necessity" of the conclusion is only the same as that of any other syllogism: If A is B, C is D; A is B; therefore C is D. Here the possibility of C being D, combined with the assertion that A *is* B, gives the necessary conclusion that C is D, which follows apodictically from the premises in the ordinary way.

But how shall we obtain the opposite of necessity—namely, *contingency*—from impossibility and non-existence combined? We see no way at all (and certainly Kant has indicated none) in which the conception of contingency, which is closely allied to that of possibility—the possibility of an event really happening—can arise out of the union of the conceptions of impossibility and non-existence: German philosophy had not, up to the time of Kant, attained to a dialectic quite so subtile and Hegelian as thus to transmute nothings into something. Our author himself has not even intimated that the category of modality was to be dealt with in the way of syllogism, in order to illustrate the general principle that each third sub-category arises from the combination of the other two; yet it is in this way only, so far as we see, that the third sub-category can be obtained, at least in the case of the second members, in the Table of Modality. We offer, therefore, the following argument, which is founded on the laws of hypothetical (conjunctive) propositions: If A is B, C cannot be D (impossibility); A is not B (non-existence), therefore C may or may not be D (contingency); in this way, contingency results, in the conclusion—if conclusion it may by courtesy be called, where formally there is none—and this result arises from the combination of the premises.*

We must defer, for the present, Kant's further remarkable developments of his Categories, and any criticisms we may have to offer on them, and on his doctrine of the understanding in general.

* The reader who is ever so slightly imbued with logic, hardly needs to be reminded, that, in conjunctives, if the antecedent be granted, the consequent is inferred (*modus ponens*); and if the consequent be denied, which is the same thing as granting its contradictory, the contradictory of the antecedent is inferred (*modus tollens*): but the affirmation of the consequent, or the denial of the antecedent, authorizes us to infer neither of the alternatives. For instance—to give a familiar illustration, which will speak for itself: from saying of a man: *if he has a fever, he is ill;* but *he is ill;* we cannot infer that *he has a fever,* for he may be ill from some other disorder. And from saying, *if he has a fever, he is ill;* but *he has not a fever;* we cannot infer that *he is not ill,* for the same reason. In the former case, he may have a fever or not, for anything that the premises contain; in the latter, he may be ill or not—all is left in uncertainty and contingence. The latter example corresponds with the one in the text.

